# The two correlations between Academic Stress and Subjective Academic Achievement: the mediating roles of Emotion Regulation and Cognitive Decline, and the moderating role of Mind Wandering

**DOI:** 10.3389/fpsyg.2026.1777509

**Published:** 2026-06-17

**Authors:** Caixia Xu, Hongliang Ma

**Affiliations:** College of Education Science, Harbin Normal University, Harbin, China

**Keywords:** academic stress, cognitive decline, emotion regulation, mind wandering, subjective academic achievement

## Abstract

This study, based on a sample of 705 social science doctoral students in China, constructs a dual-mediation model of “Academic Stress → Emotion Regulation/Cognitive Decline → Subjective Academic Achievement” and introduces Mind Wandering as a moderating variable to examine the two correlational pathways between Academic Stress and Subjective Academic Achievement. The findings reveal that: (1) Academic Stress is positively correlated with Emotion Regulation (*B* = 0.322, *p* < 0.001), and Emotion Regulation is positively correlated with Subjective Academic Achievement (*B* = 0.137, *p* < 0.001). Concurrently, in the negative pathway, Academic Stress is positively correlated with Cognitive Decline (*B* = 0.335, *p* < 0.001), and Cognitive Decline is negatively correlated with Subjective Academic Achievement (*B* = −0.126, *p* < 0.001). (2) Mind Wandering significantly moderates both pathways: under conditions of high Mind Wandering, the positive correlation between Academic Stress and Emotion Regulation is stronger, and the positive correlation between Academic Stress and Cognitive Decline is also stronger. This study reveals the potential “double-edged sword effect” of Academic Stress through the parallel mediating roles of Emotion Regulation and Cognitive Decline, and validates the double-edged moderating mechanism of Mind Wandering on the stress-transformation pathways. The findings provide theoretical foundations and practical guidance for universities to design differentiated stress intervention programs.

## 1 Introduction

As a core indicator of doctoral training quality, academic achievement is consistently shaped by the interplay between individual psychological factors and environmental pressures. In recent years, with intensifying competition in higher education, doctoral students have faced multiple stressors, including heavy research workloads, high publication demands, and heightened career uncertainty (Evans, 2018). Existing research presents two opposing perspectives—beneficial and detrimental—regarding the relationship between Academic Stress and academic achievement, reflecting the context-dependent nature of stress effects and the complexity of underlying mediating mechanisms. Academic Stress does not influence academic performance through a simple linear pathway; rather, it may exert a complex “double-edged sword effect” through two indirect pathways: Emotion Regulation and cognitive function. On one hand, moderate stress can motivate intrinsic Emotion Regulation, prompting individuals to adopt adaptive strategies such as Cognitive Reappraisal to enhance academic engagement (Bi et al., 2025). On the other hand, chronic excessive stress may deplete cognitive resources, leading to attentional lapses and reduced creativity (Wang et al., 2019).

Existing research has two major limitations: First, most relevant studies treat emotion regulation as an explanatory variable for academic performance or achievement (Romo et al., 2025), overlooking the potential role of Emotion Regulation in the relationship between Academic Stress and Academic Achievement. Second, investigations into indirect pathways are mostly limited to single-mediator models, lacking comparative studies on the parallel roles of emotion regulation and cognitive function. Academic achievement among doctoral students is difficult to assess using grades. Previous research has found that Subjective Academic Achievement scales are highly correlated with objective measures of academic achievement (Pike, 1995; Felder-Puig et al., 2012). Therefore, this study will measure participants' academic achievement using a subjective self-report scale (Stadler et al., 2021).

Previous research on the relationship between Academic Stress and Emotion Regulation has yielded two conclusions. The first suggests that “Emotion Regulation reduces Academic Stress” (Iuga and David, 2024; Kritikou and Giovazolias, 2022), while the second indicates that “Academic Stress triggers Emotion Regulation” (Sang et al., 2018). Analysis of the questionnaire data in this study shows that the Chinese doctoral student sample aligns with the second pattern. Qualitative interviews explain why this pattern emerged, with differences in Cognitive Reappraisal orientations being the primary reason. Upward and Downward Cognitive Reappraisal differ in their goals, leading to two opposite correlations between Cognitive Reappraisal and Academic Stress. This study will explore the relationship between different types of reappraisal orientations and Academic Stress, as well as the reappraisal orientations of Chinese doctoral students in a high-pressure academic environment.

The Expressive Suppression dimension of Emotion Regulation can enhance interpersonal relationships (Gross and John, 2003). Interpersonal relationships, in turn, have a potential impact on Academic Achievement (Yang et al., 2023). For doctoral students, vertical supervisor-student relationships and horizontal peer relationships may be associated with their Academic Achievement. Accordingly, a potential pathway of “Academic Stress → Expressive Suppression → Interpersonal Relationships → Academic Achievement” may exist between Academic Stress and Academic Achievement. This study will explore these potential correlations using survey data and qualitative research.

Furthermore, Academic Stress may negatively affect Academic Achievement, and this study will examine the underlying mechanism of Cognitive Decline in this relationship.

Focusing on Chinese doctoral students, this study constructs a dual-mediation model of “Academic Stress → Emotion Regulation/Cognitive Decline → Subjective Academic Achievement.” Theoretically, these two pathways represent two key mechanisms linking Academic Stress and Subjective Academic Achievement, reflecting the positive and negative dimensions of Academic Stress, respectively. Mind Wandering, a common psychological phenomenon under stress, is characterized by the drift of attention from an ongoing task to internal thoughts (Smallwood et al., 2007). In the context of Academic Stress, Mind Wandering may cause individuals to more frequently become immersed in stress-related rumination or self-focused attention, thereby intensifying the activation of Emotion Regulation strategies (such as Cognitive Reappraisal and Expressive Suppression) by stress. Concurrently, Mind Wandering may also amplify the depletion of cognitive resources by stress. Therefore, this study will investigate the potential moderating role of Mind Wandering in the two pathways mentioned above.

This study seeks to address the following questions: (1) How is Academic Stress correlated with Subjective Academic Achievement through the two pathways of Emotion Regulation and Cognitive Decline? (2) Does Mind Wandering strengthen or weaken the correlations in these pathways? (3) For individuals in extremely high-stress environments, is the correlation between Academic Stress and Emotion Regulation positive or negative? The findings will not only enrich the application of stress-coping theories in higher education contexts but also provide empirical evidence to inform mental health interventions and academic support systems for doctoral students.

### 1.1 Literature review and theoretical hypotheses

#### 1.1.1 Academic stress and academic achievement

Scholars hold two perspectives on the relationship between Academic Stress and Academic Achievement: Academic Stress reduces Academic Achievement, or Academic Stress can promote Academic Achievement under specific conditions.

Academic Stress may indirectly affect Academic Achievement through anxiety and school burnout. Chronic high stress leads to issues such as sleep disturbances and attentional lapses in students, directly impacting learning efficiency (Wang and Fan, 2023). Academic Stress may negatively affect academic performance, while active coping strategies can mitigate the negative impact of stress on academic outcomes (Basith et al., 2021). Gustems-Carnicer et al. (2019) proposed that for students in teacher education programs, perceived Academic Stress is significantly negatively correlated with Academic Achievement. Liu and Lu (2011) found that Academic Stress significantly negatively predicted the Academic Achievement of a portion (87%) of participating students, but had no significant effect on the remaining (13%) students.

García-Martínez et al. (2021) proposed that the impact of Academic Stress on educational achievement may be indirectly mediated through the moderating role of emotional intelligence. Students with high emotional intelligence can manage stress more effectively, reducing the negative impact of Academic Stress on achievement. Yuda et al. (2022) suggested that students with high self-efficacy can cope with stress more proactively, thereby maintaining higher Academic Achievement. Active coping strategies are significantly positively correlated with high Academic Achievement, whereas passive coping may exacerbate the negative effects of stress. Oyewobi et al. (2021) proposed that moderate stress (e.g., exam pressure) can motivate undergraduate students and enhance academic performance, whereas excessive stress (e.g., chronic anxiety) impairs their cognitive function, leading to decreased performance. Shih and Lin (2017) demonstrated that at lower levels of academic anxiety, academic anxiety enhances academic performance; at high levels of academic anxiety, this positive association is not significant. Zhu et al. (2017) found that challenge-related stress (e.g., workload) is positively associated with Academic Achievement, whereas hindrance-related stress (e.g., role ambiguity) is negatively associated with Academic Achievement.

The above literature review indicates that there may be both positive and negative correlations between Academic Stress and Academic Achievement. When examining the relationship between the two, it is necessary to consider both positive and negative scenarios.

### 1.2 Academic stress and emotion regulation

Regarding the correlation between Academic Stress and Emotion Regulation, two opposing conclusions exist. The first suggests a negative correlation between Emotion Regulation and Academic Stress, indicating that Emotion Regulation can reduce Academic Stress. The second suggests a positive correlation between the two. Previous research has predominantly supported the first conclusion (Extremera and Rey, 2015; O'Day et al., 2019; Berking et al., 2008). In these studies, Emotion Regulation serves as an explanatory variable, defined as a psychological intervention or the capacity and effectiveness of regulating emotions.

However, does Academic Stress prompt individuals to engage in Emotion Regulation? Stress typically motivates individuals to adopt adaptive coping strategies based on the context (Spătaru et al., 2024). Stress coping refers to the efforts individuals make to manage or adapt to stressful events, encompassing both behavioral and psychological strategies (Folkman and Lazarus, 1986). Stress prompts individuals to select coping styles that align with cultural norms. Individuals from different cultural backgrounds exhibit significant differences in how they cope with stress (See and Essau, 2010). During the COVID-19 pandemic, psychotherapists used self-care to alleviate occupational stress (Rokach and Boulazreg, 2022). Exam stress can trigger Emotion Regulation motivation among Chinese adolescents. Before examinations, students may use regulation to amplify positive emotions to enhance confidence or suppress negative emotions to alleviate exam stress (Sang et al., 2018).

As noted above, previous research has often shown a negative correlation between Academic Stress and Emotion Regulation—that is, “Emotion Regulation reduces Academic Stress.” However, at most Chinese universities, publishing papers in SCI, SSCI, or CSSCI^1^ is a mandatory requirement for graduation. Under such extreme academic pressure, doctoral students' understanding and coping with stress may be unique. To achieve higher Academic Achievement, Chinese doctoral students may adopt Achievement-Oriented Cognitive Reappraisal, transforming Academic Stress into academic motivation. In this process, doctoral students are less concerned with eliminating stress and may even actively impose or maintain pressure on themselves to sustain motivation. Additionally, Academic Stress requires doctoral students to maintain positive academic interpersonal relationships. Supervisors' guidance and peers' academic collaboration can contribute to doctoral students' Academic Achievement.

Based on the above rationale, we propose:

- **Hypothesis 1:** Academic Stress is positively correlated with Emotion Regulation.

### 1.3 Emotion regulation and subjective academic achievement

The positive effect of Emotion Regulation on Academic Achievement is widely recognized (Graziano et al., 2007; Djambazova-Popordanoska, 2016; Xu et al., 2019; Gelhaar et al., 2007).

Two key dimensions of Emotion Regulation—Cognitive Reappraisal and Expressive Suppression—have potential positive effects on Academic Achievement (Balzarotti et al., 2017; Jones and Destin, 2021; Folkman and Moskowitz, 2004).

Emotion Regulation can promote positive emotions, which in turn enhance creativity by facilitating cognitive flexibility (De Dreu et al., 2024). Emotion Regulation contributes to the formation of an open personality, which is a significant predictor of creativity (Karwowski et al., 2021).

Cognitive Reappraisal in academic contexts can be either Achievement-oriented or Stress Reduction-oriented. That is, individuals can enhance Academic Achievement through Upward Cognitive Reappraisal (e.g., self-motivation, aspiration, upward meaning reconstruction) or reduce Academic Achievement through Downward Cognitive Reappraisal (e.g., lowering expectations, downward reconstruction of learning goals). Therefore, this study proposes the innovative concepts of Upward Cognitive Reappraisal and Downward Cognitive Reappraisal. In high-pressure environments, individuals may adopt Stress Reduction-oriented (Downward) Cognitive Reappraisal to alleviate stress. When high pressure coexists with high Academic Achievement motivation, doctoral students may adopt Achievement-oriented (Upward) Cognitive Reappraisal.

Furthermore, Expressive Suppression has relatively fewer negative effects on East Asians (Chen et al., 2020). In high academic pressure environments, Chinese doctoral students may utilize Expressive Suppression to enhance vertical supervisor-student relationships and horizontal peer relationships. For relationships that cannot be directly captured in survey data, this study will employ qualitative interviews to explore them.

Based on the above rationale, the following hypotheses are proposed:

- **Hypothesis 2:** Emotion Regulation among the Chinese doctoral students is positively correlated with subjective Academic Achievement.
- **Hypothesis 3:** Emotion Regulation mediates the relationship between Academic Stress and subjective Academic Achievement among the Chinese doctoral students.

### 1.4 Academic stress, cognitive decline and subjective academic achievement

The negative effects of Academic Stress on cognition and Academic Achievement are widely recognized.

Chronic exposure to stress can lead to damage to neurons, cognition, and memory (McEwen and Sapolsky, 1995). High stress levels may negatively affect students' physical health, which in turn may impact Academic Achievement (Rafique et al., 2020; Stewart et al., 2018). Jung et al. (2015) proposed that Academic Stress indirectly exacerbates academic burnout by reducing academic self-efficacy, thereby impairing students' cognitive executive functions. Yuhuan et al. (2022) proposed that Academic Stress weakens students' cognitive control abilities (such as impulse inhibition and delayed gratification) through self-regulatory fatigue, consequently affecting learning efficiency. Vogel and Schwabe (2016) pointed out that stress during learning may enhance memory formation but significantly impairs memory retrieval, leading to poor exam performance. Additionally, stress may shift learning from flexible cognitive forms to rigid habit-based forms.

Based on the above rationale, the following hypotheses are proposed:

- **Hypothesis 4:** Academic Stress is positively correlated with Cognitive Decline.
- **Hypothesis 5:** Cognitive Decline is negatively correlated with subjective Academic Achievement.
- **Hypothesis 6:** Cognitive Decline mediates the relationship between Academic Stress and subjective Academic Achievement.

### 1.5 Mind wandering and its moderating role

Mind Wandering can be viewed as a goal-driven process, although this process is not directed toward the current primary task (Smallwood and Schooler, 2006). The impact of Mind Wandering on tasks depends on various factors, including the nature of the task and the individual's psychological state (Gericke et al., 2022). Whether Mind Wandering improves mood depends on two conditions: whether Mind Wandering is accompanied by meta-awareness and whether Mind Wandering occurs in a state of high alertness or low alertness (Konjedi and Maleeh, 2017). If the content of Mind Wandering is negative in nature, individuals feel worse after Mind Wandering; if the content is positive, mood does not decline (Poerio et al., 2013). During Mind Wandering, positive emotions decrease among participants under positive and neutral mood conditions; negative emotions also decrease among participants under negative mood conditions (Nayda and Takarangi, 2025).

Mind Wandering is beneficial to divergent thinking and creative thinking, and these benefits have potential associations with Cognitive Reappraisal during Emotion Regulation (Rodriguez-Boerwinkle et al., 2024; Garland et al., 2015). Theoretically, Mind Wandering helps doctoral students break fixed perceptions of emotional events, thereby initiating and reinforcing Cognitive Reappraisal.

As discussed in Sections 1.2, 1.3, theoretically, doctoral students need to enhance interpersonal relationships through Expressive Suppression, thereby gaining opportunities to improve Academic Achievement (supervisor assistance, team collaboration). In the context of Academic Stress, Mind Wandering can provide graduate students with a psychological “escape,” allowing individuals to temporarily detach from stressors (Welhaf et al., 2024). By releasing suppressed emotions through internal thinking or fantasy, these “escapes” can theoretically help doctoral students better inhibit negative emotional expression in interpersonal contexts.

Based on the above rationale, the following hypothesis is proposed:

- **Hypothesis 7:** Mind Wandering positively moderates the correlation between Academic Stress and Emotion Regulation.

Positive-constructive Mind Wandering promotes task-related thinking, whereas poor-attention Mind Wandering promotes task-unrelated thinking (Soemer et al., 2024). Mind Wandering has a dual impact on creativity and mental health, potentially promoting creative problem-solving on one hand, while leading to negative emotions and mental health issues on the other (Yamaoka and Yukawa, 2020). Mind Wandering has a significant negative impact on working memory capacity and intelligence test performance, and is associated with negative emotions, potentially increasing stress and reducing life satisfaction (Mooneyham and Schooler, 2013). Mind Wandering may reduce task focus, processing efficiency, and accuracy. Mind Wandering reduces the brain's rest time, and excessive Mind Wandering can increase cognitive fatigue and reduce cognitive function (Zhang and Kool, 2026). Under conditions of Academic Stress, Mind Wandering may serve as an “avoidance mechanism,” allowing graduate students to temporarily alleviate cognitive tension by diverting attention when facing Academic Stress. However, excessive avoidance may exacerbate the dispersion of cognitive resources, leading to Cognitive Decline (Morrison et al., 2014).

**Figure 1:**
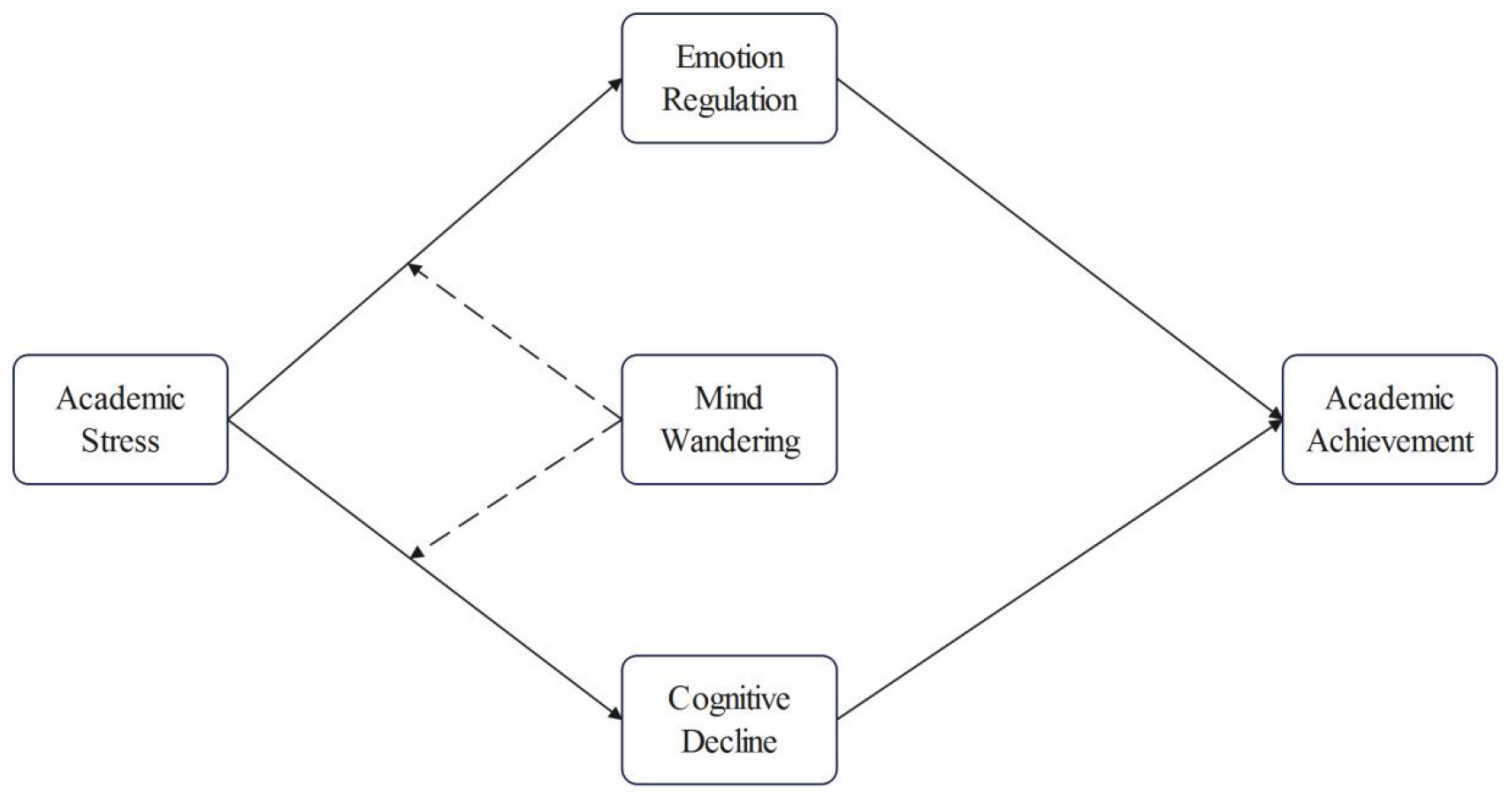
Schematic diagram of hypotheses.

Based on the above rationale, the following hypothesis is proposed:

- **Hypothesis 8:** Mind Wandering positively moderates the correlation between Academic Stress and Cognitive Decline. The schematic diagram of the hypotheses is shown in Figure 1.

## 2 Research methods

### 2.1 Research sample

This study employed a questionnaire survey to collect data, with the data sourced from Harbin City, Heilongjiang Province, China. Harbin is one of the educational center cities in Northeast China, home to 49 universities. The respondents comprised social science doctoral students enrolled in Harbin between 2022 and 2023. Questionnaires were distributed through both online and offline channels.

To mitigate the potential impact of common method bias, data collection employed a time-lagged approach conducted in two phases. The first phase collected respondents' demographic information, Academic Stress questionnaire, and Mind Wandering questionnaire. The second phase collected respondents' Emotion Regulation questionnaire, Cognitive Decline questionnaire, and subjective Academic Achievement questionnaire. The two phases were separated by a 4-week interval. After excluding incomplete questionnaires, 705 valid responses were obtained, yielding an effective response rate of 75.3%.

### 2.2 Variable measurement

All scales used in this study were well-established instruments. All questionnaires were processed using a “translation-back translation” procedure to ensure accuracy and comprehensibility of the content. Academic Stress, Emotion Regulation, Cognitive Decline, Mind Wandering, and Subjective Academic Achievement were all measured using a 7-point Likert scale.

Academic Stress was measured using *the Perceptions of Academic Stress Scale* developed by Bedewy and Gabriel (2015). This scale consists of 18 items measuring three dimensions: academic expectations, faculty work and examinations, and academic self-perceptions. The Cronbach's α value for this scale in the present study was 0.928.

Emotion Regulation was measured using *the Emotion Regulation Questionnaire* developed by Gross and John (2003). This scale consists of 10 items measuring two dimensions: reappraisal and suppression. This scale has been widely used to measure Emotion Regulation (Gómez-Ortiz et al., 2016; Hutchison et al., 2021; Chang, 2020). The Cronbach's α value for this scale in the present study was 0.904.

Cognitive Decline was measured using *the Cognitive Failures Questionnaire* developed by Broadbent et al. (1982). This scale consists of 25 items. It does not measure organic cognitive impairment but rather assesses the degree of Cognitive Decline across three dimensions: perception, memory, and attention. The Cronbach's α value for this scale in the present study was 0.949.

Mind Wandering was measured using *the Mind-Wandering Questionnaire* developed by Mrazek et al. (2013). This scale consists of 12 items focusing on the frequency of Mind Wandering. The items employ neutral wording, reflecting the objective phenomenon of attention drifting away from the task. This scale is therefore suitable for measuring the double-edged sword effect of Mind Wandering in this study. The Cronbach's α value for this scale in the present study was 0.904.

Subjective Academic Achievement was measured using *the Subjective Academic Achievement Scale* (Stadler et al., 2021). This scale consists of five items assessing respondents' Subjective Academic Achievement across five dimensions: grade satisfaction, sense of academic success, learning efficiency, academic progress, and peer comparison. The Cronbach's α value for this scale in the present study was 0.897.

Based on findings from previous research (Yau et al., 2025; Shu et al., 2025; Cao and Mithra, 2024), gender, annual household income, and hometown type were selected as control variables. For gender, 0 represented female and 1 represented male. Annual household income was measured using a 6-point scale, with 1–6 corresponding to ≤ 20,000 RMB, 20,001–40,000 RMB, 40,001–60,000 RMB, 60,001–80,000 RMB, 80,001–100,000 RMB, and >100,000 RMB, respectively. For hometown type, 0 represented urban hometown and 1 represented rural hometown.

## 3 Data analysis

### 3.1 Common method bias test

The Harman single-factor method was used to test for common method bias. The results showed that the variance explained by the first common factor was 22.216%, which was below 40%. Additionally, after adding a method factor to the original factor structure, the common method bias was tested again, and the results indicated that the model fit indices did not significantly improve after the inclusion of the method factor. This suggests that common method bias is not a serious concern in this study.

### 3.2 Confirmatory factor analysis

Confirmatory factor analysis was conducted to examine the discriminant validity of Academic Stress, Emotion Regulation, Cognitive Decline, Mind Wandering, and Subjective Academic Achievement. The results showed that the five-factor model demonstrated the best fit (χ^2^/df = 1.199, RMSEA = 0.017, GFI = 0.901, CFI = 0.979, NNFI = 0.979). This indicates good discriminant validity among the five subjectively evaluated variables in this study (Table 1).

**Table 1:** Results of the confirmatory factor analysis.

Model	*χ^2^*/df	RMSEA	GFI	CFI	NNFI
Five-factor	1.199	0.017	0.901	0.979	0.978
Four-factor	2.695	0.049	0.659	0.819	0.813
Three-factor	4.081	0.066	0.478	0.67	0.66
Two-factor	5.154	0.077	0.409	0.555	0.541
Single-factor	5.978	0.084	0.386	0.466	0.45

Five-factor model: AS, ERM, CD, MW, AA; Four-factor model: AS+MW, ERM, CD, AA; Three-factor model: AS+ERM+CD, MW, AA; Two-factor model: AS+ERM+CD+MW, AA; Single-factor model: AS+MW+ERM+CD+AA.

### 3.3 Descriptive statistics and correlation analysis

Descriptive statistics and correlation analysis revealed that Academic Stress was significantly positively correlated with Emotion Regulation, Academic Stress was significantly positively correlated with Cognitive Decline, Emotion Regulation was significantly positively correlated with Subjective Academic Achievement, and Cognitive Decline was significantly negatively correlated with Subjective Academic Achievement. Thus, the research hypotheses received preliminary support.

Gender was significantly positively correlated with Academic Stress, which may be related to male doctoral students' future financial responsibilities and pressures, with male doctoral students experiencing greater Academic Stress than their female counterparts. Gender was significantly negatively correlated with Emotion Regulation, indicating that male doctoral students in this study used Emotion Regulation less frequently. Previous research has shown diverse correlations between Emotion Regulation and gender (Perchtold et al., 2019; Extremera and Rey, 2015). Family income was significantly correlated with Academic Stress, with doctoral students from higher-income families reporting relatively lower Academic Stress. Additionally, China's urban-rural economic development disparities contributed to the correlation between household registration type (0 representing urban, 1 representing rural) and family income, as well as the correlation between household registration type and Academic Stress.

### 3.4 Hypothesis testing

#### 3.4.1 Mediation effect testing

The mediation effects were tested using Mplus 8.3 (Muthén & Muthén, Los Angeles, CA, USA) software, and the results of the bootstrap method with 5,000 resamples showed:

In Model 3, the total effect between Academic Stress and Subjective Academic Achievement was significant (*B* = 0.125, *p* < 0.01). This indicates that for the doctoral students in this sample, the overall correlation between Academic Stress and Subjective Academic Achievement was positive.

In Model 1, Academic Stress was significantly positively correlated with Emotion Regulation (*B* = 0.322, SE = 0.033, *p* < 0.001). In Model 4, Emotion Regulation was significantly positively correlated with Subjective Academic Achievement (*B* = 0.137, SE = 0.041, *p* < 0.001). In Model 6, Emotion Regulation was significantly positively correlated with Subjective Academic Achievement (*B* = 0.104, SE = 0.043, *p* < 0.05). The mediation effect of Emotion Regulation between Academic Stress and Subjective Academic Achievement was 0.033, with a 95% CI = (0.006, 0.063), which did not contain zero. In Hypothesis Path 1, the direct effect between Academic Stress and Subjective Academic Achievement was significant (*B* = 0.091, SE = 0.041, *p* < 0.05), with a 95% CI = (0.011, 0.072), which did not contain zero. Therefore, Hypotheses 1, 2, and 3 were supported.

In Model 2, Academic Stress was significantly positively correlated with Cognitive Decline (*B* = 0.335, SE = 0.043, *p* < 0.001). In Model 5, Cognitive Decline was significantly negatively correlated with Subjective Academic Achievement (*B* = −0.126, SE = 0.033, *p* < 0.001). In Model 7, Cognitive Decline was significantly negatively correlated with Subjective Academic Achievement (*B* = −0.170, SE = 0.033, *p* < 0.001). The mediation effect of Cognitive Decline between Academic Stress and Subjective Academic Achievement was −0.057, with a 95% CI = (−0.086, −0.031), which did not contain zero. In Hypothesis Path 2, the direct effect between Academic Stress and Subjective Academic Achievement was significant (*B* = 0.182, SE = 0.039, *p* < 0.001), with a 95% CI = (0.104, 0.259), which did not contain zero. Therefore, Hypotheses 4, 5, and 6 were supported.

#### 3.4.2 Moderation effect testing

As shown in Table 2, the interaction term between Academic Stress and Mind Wandering was significantly correlated with Emotion Regulation (*B* = 0.079, *p* < 0.01). The interaction term between Academic Stress and Mind Wandering was significantly correlated with Cognitive Decline (*B* = 0.100, *p* < 0.01). Therefore, Hypotheses 7 and 8 were supported.

**Table 2:** Regression analysis of moderation effects.

variable	Emotion regulation	Cognitive decline
Gender	−0.207^***^	0.031
Household registration	0.04	0.023
Family income	−0.016	0.006
Academic stress	0.329^***^	0.344^***^
Mind wandering	0.303^***^	0.362^***^
Academic stress × mind wandering	0.079^**^	0.1^**^
*R^2^*	0.318	0.256
*F*	54.316^***^	40.041^***^

^**^p < 0.01, ^***^p < 0.001.

**Figure 2:**
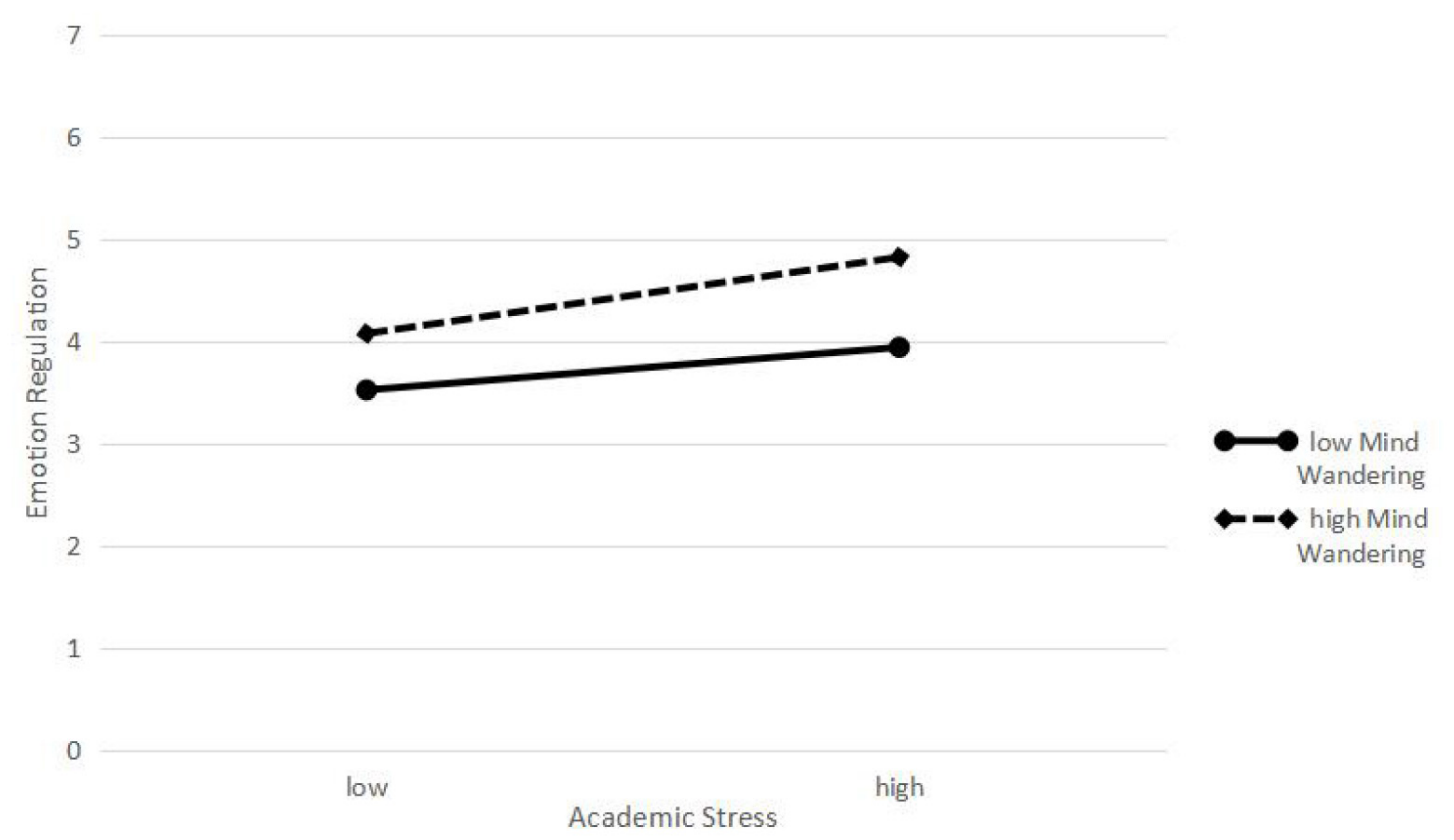
Simple slope plot of the moderating effect of mind wandering on the relationship between academic stress and emotion regulation.

**Figure 3:**
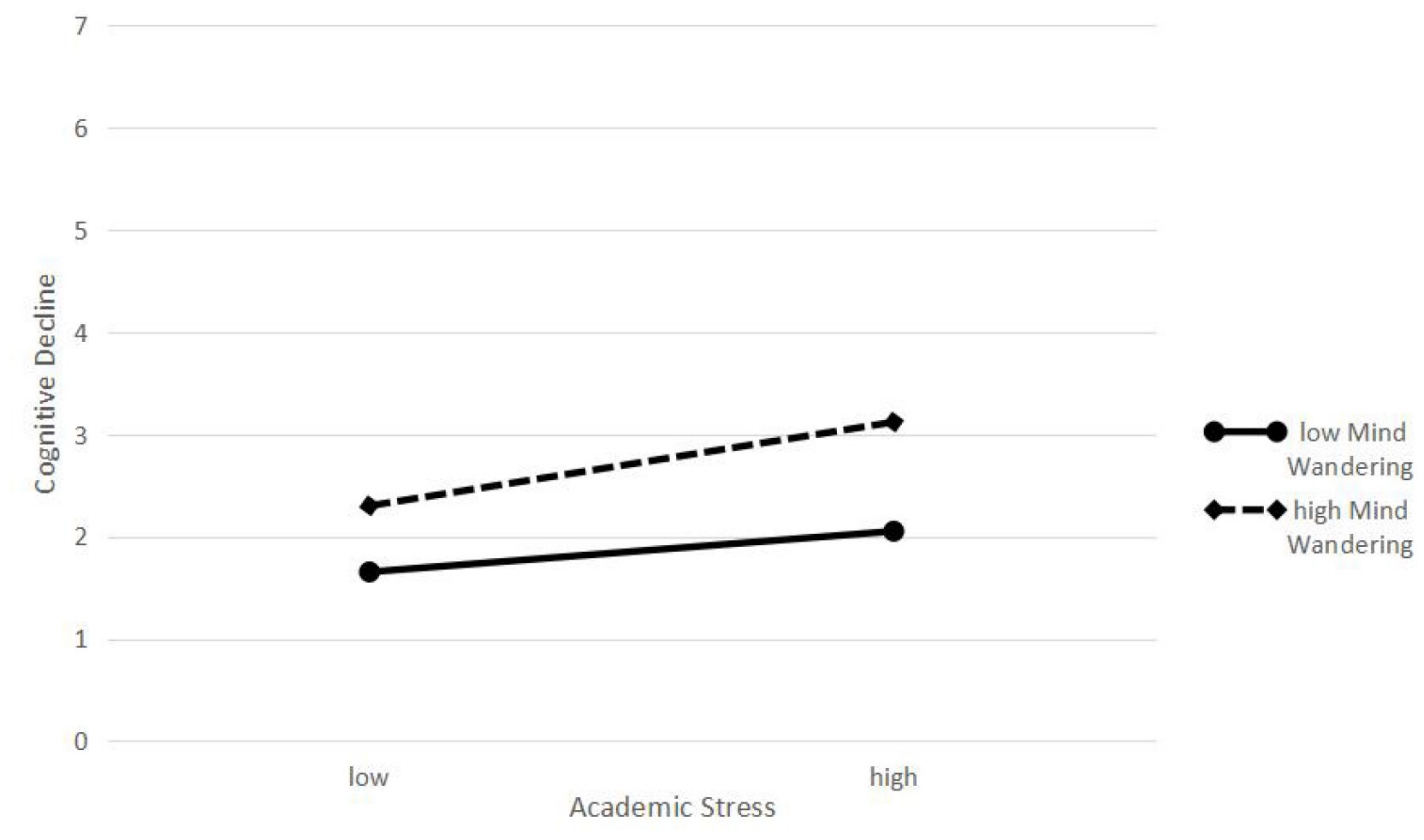
Simple slope plot of the moderating effect of mind wandering on the relationship between academic stress and cognitive decline.

To more intuitively illustrate the moderation effects, moderation plots were drawn based on one standard deviation above the mean and one standard deviation below the mean (see Figures 2, 3). As shown in Figures 2, 3, under conditions of high Mind Wandering, the positive correlation between Academic Stress and Emotion Regulation was stronger, and the positive correlation between Academic Stress and Cognitive Decline was also stronger.

Further testing of the moderated mediation effects was conducted. The results showed that under the condition of one standard deviation below the mean of Mind Wandering, the indirect correlation between Academic Stress and Subjective Academic Achievement through Emotion Regulation was 0.024, with a 95% CI = (0.003, 0.05), which did not contain zero. Under the condition of one standard deviation above the mean of Mind Wandering, the indirect correlation between Academic Stress and Subjective Academic Achievement through Emotion Regulation was 0.044, with a 95% CI = (0.008, 0.081), which did not contain zero. The difference in the indirect correlation between the two conditions was 0.02, with a 95% CI = (0.001, 0.043), which did not contain zero. Therefore, Mind Wandering significantly moderated the indirect correlation between Academic Stress and Subjective Academic Achievement through Emotion Regulation.

Under the condition of one standard deviation below the mean of Mind Wandering, the indirect correlation between Academic Stress and Subjective Academic Achievement through Cognitive Decline was −0.038, with a 95% CI = (−0.063, −0.018), which did not contain zero. Under the condition of one standard deviation above the mean of Mind Wandering, the indirect correlation between Academic Stress and Subjective Academic Achievement through Cognitive Decline was −0.078, with a 95% CI = (−0.118, −0.043), which did not contain zero. The difference in the indirect correlation between the two conditions was −0.04, with a 95% CI = (−0.073, −0.011), which did not contain zero. Therefore, Mind Wandering significantly moderated the indirect correlation between Academic Stress and Subjective Academic Achievement through Cognitive Decline.

## 4 Qualitative study on “academic stress → emotion regulation → subjective academic achievement”

To further explore the relationship between Academic Stress and Emotion Regulation, volunteers were recruited from the doctoral students who had completed the aforementioned questionnaire survey. Prior to the interviews, participants were briefly introduced to the basic concepts of Cognitive Reappraisal and Expressive Suppression. A total of 35 individuals were interviewed, of whom 32 reported qualitative content regarding their Academic Stress and Emotion Regulation. The remaining three individuals indicated that they did not experience relevant psychological or behavioral phenomena.

The interview data from the 32 participants were coded using NVivo 22.0 (QSR International Pty Ltd, Melbourne, Victoria, Australia) software. First, during the open coding stage, the interview data were analyzed and conceptualized, with duplicate labels merged, to preliminarily generate first-level conceptual labels. Second, the meanings of the concepts were clarified, and relationships among similar concepts were compared. Finally, the relational structures among the categories were further specified, and selective coding was conducted to derive the two categories: “Cognitive Reappraisal” and “Expressive Suppression.”

Regarding Cognitive Reappraisal, there exist two opposing reappraisal orientations. Among the 32 individuals, 29 expressed Upward Cognitive Reappraisal, while the remaining three were categorized as exhibiting Downward Cognitive Reappraisal. These 29 individuals demonstrated stronger subjective academic pressure, academic expectations, or Academic Achievement.

In Chinese universities, a vertical obedience relationship commonly exists between supervisors and students. This relationship necessitates that doctoral students strictly employ Expressive Suppression toward their supervisors. Even when dissatisfied with their supervisors, they must suppress such feelings and express as many positive emotions as possible. Furthermore, the interview content indicated that horizontal peer relationships are very important for doctoral students' campus living environment and team collaboration.

These qualitative findings further explain the correlations among Academic Stress, Emotion Regulation, and Academic Achievement presented in Sections 3.3, 3.4, as well as why these correlations differ from earlier research conclusions (Iuga and David, 2024; Kritikou and Giovazolias, 2022). Under conditions of immense academic pressure, the majority of doctoral students opted for Upward Cognitive Reappraisal and Expressive Suppression. At the same time, upward reappraisal may intensify Academic Stress. Upward Cognitive Reappraisal and Expressive Suppression may promote Academic Achievement. Reappraisal orientation is an important factor determining the relationship between Academic Stress and Emotion Regulation. For the Chinese doctoral students in this sample, Downward Cognitive Reappraisal may negatively affect Academic Stress and Academic Achievement. Although other studies have identified a pathway whereby “Emotion Regulation reduces Academic Stress, thereby enhancing Academic Achievement” (Wang and Wang, 2025; Journault et al., 2025), the results of this study suggest that Chinese doctoral students tend to transform Academic Stress into Emotion Regulation (Upward Cognitive Reappraisal and Expressive Suppression), thereby enhancing Academic Achievement.

## 5 Conclusions and recommendations

### 5.1 Discussion

This study reveals the correlations between Academic Stress and Subjective Academic Achievement through the parallel mediating pathways of Emotion Regulation and Cognitive Decline, and verifies the moderating role of Mind Wandering on both pathways.

Academic Stress is correlated with subjective Academic Achievement through two competing pathways:

Positive pathway: Academic Stress is positively correlated with Emotion Regulation (*B* = 0.322, *p* < 0.001), Emotion Regulation is positively correlated with Subjective Academic Achievement (*B* = 0.137, *p* < 0.001), and the mediation effect value of Emotion Regulation is 0.033.

Negative pathway: Academic Stress is positively correlated with Cognitive Decline (*B* = 0.335, *p* < 0.001), Cognitive Decline is negatively correlated with Subjective Academic Achievement (*B* = −0.126, *p* < 0.001), and the mediation effect value of Cognitive Decline is −0.057.

Mind Wandering significantly moderates both pathways:

Under conditions of high Mind Wandering, the positive correlation between Academic Stress and Emotion Regulation is stronger. Concurrently, under conditions of high Mind Wandering, the positive correlation between Academic Stress and Cognitive Decline is also stronger.

Academic Stress is prevalent among students across countries worldwide, and how to cope with Academic Stress represents an important research direction. Employing appropriate strategies can transform stress into Academic Achievement. In Table 3, Academic Stress is significantly positively correlated with subjective Academic Achievement (*r* = 0.115, *p* < 0.01). In Table 4, Model 3 shows a significant regression result between Academic Stress and Subjective Academic Achievement (*B* = 0.125, *p* < 0.01). This indicates that for Chinese doctoral students, the overall relationship between Academic Stress and Subjective Academic Achievement is positive, meaning that the positive correlation between the two far outweighs the negative correlation.

**Table 3:** Descriptive statistics and correlation analysis results.

variables	mean	SD	Gender	Household registration	Family income	Academic stress	Emotion regulation	Cognitive decline	Mind wandering
Gender	0.494	0.5							
Household registration	0.309	0.463	0.045						
Family income	4.288	1.195	0.009	−0.105^**^					
Academic stress	3.676	0.886	0.077^*^	0.085^*^	−0.089^*^				
Emotion regulation	4.091	0.831	−0.099^**^	0.026	−0.058	0.336^**^			
Cognitive decline	2.278	1.036	0.029	0.013	−0.02	0.285^**^	0.383^**^		
Mind wandering	2.381	1.14	−0.014	−0.066	0.066	−0.034	0.41^**^	0.394^**^	
Subjective academic Achievement	3.284	0.902	−0.036	−0.03	−0.001	0.115^**^	0.127^**^	−0.146^**^	0.024

^*^p < 0.05, ^**^p < 0.01.

**Table 4:** Regression analysis of mediation effects.

Variable	Emotion regulation	Cognitive decline	Subjective academic achievement				
	Model 1	Model 2	Model 3	Model 4	Model 5	Model 6	Model 7
Gender	−0.207^***^	0.016	−0.079	−0.04	−0.055	−0.057	−0.076
Household registration	0.001	−0.024	−0.073	−0.062	−0.053	−0.073	−0.077
Family income	−0.018	0.004	0.005	0.003	−0.005	0.007	0.006
Academic stress	0.322^***^	0.335^***^	0.125^**^			0.091^*^	0.182^***^
Emotion regulation				0.137^***^		0.104^*^	
Cognitive decline					−0.126^***^		−0.17^***^
Mind wandering							
*R^2^*	0.129	0.082	0.017	0.018	0.023	0.025	0.052
*F*	25.905^***^	15.563^***^	2.992^*^	3.161^*^	4.131^**^	3.545^**^	7.628^***^

^*^p < 0.05, ^**^p < 0.01, ^***^p < 0.001.

Regarding the positive effects of Academic Stress on Academic Achievement, previous research has focused on potential mechanisms such as task focus (Saklofske et al., 2012) and learning efficacy (Chongjin et al., 2025). This study demonstrates an additional mechanism: Emotion Regulation.

Regarding the relationship between Emotion Regulation and Academic Stress, two opposing perspectives exist: “regulation can reduce Academic Stress” or “the greater the stress, the stronger the motivation to regulate.” The difference lies in whether the focus is on “eliminating stress” vs. “transforming stress.” The first type of correlation has been extensively discussed, i.e., “Emotion Regulation reduces stress” (Iuga and David, 2024; Kritikou and Giovazolias, 2022). The second perspective, however, is rarely observed. One study focusing on Chinese middle school students (Sang et al., 2018) proposed this second perspective, finding that exam stress prompted participating middle school students to use Emotion Regulation. Another study focusing on Chinese undergraduate students (Pu et al., 2024) identified a similar correlation, whereby participants' perceived stress positively influenced Cognitive Reappraisal, indirectly affecting anxiety. Further manual retrieval and GenAI analysis of studies examining both Emotion Regulation and Academic Stress revealed that in the vast majority of studies, Emotion Regulation and Academic Stress are negatively correlated. That is, in most academic contexts, Emotion Regulation is employed as a tool to alleviate Academic Stress, implying that the relevant Cognitive Reappraisal is Stress Reduction-oriented. These strategic differences may stem from variations in educational competitiveness and student psychological traits across different countries. The qualitative findings reveal that Stress Reduction-oriented Cognitive Reappraisal may include lowering expectations or downward reconstruction of the meaning of learning. Achievement-oriented Cognitive Reappraisal may include self-motivation, aspiration, and reappraisal of opportunities.

The educational environment in East Asia is highly competitive. Middle school and university students in East Asia generally face intense horizontal competition (Xu and Wen, 2025). The sample of Chinese doctoral students in this study is situated within a unique stress context. Compared with their counterparts in neighboring East Asian countries such as Japan and South Korea, Chinese graduate students face greater pressure. Publishing papers in SCI, SSCI, or China's domestic CSSCI-indexed journals is a graduation requirement for most doctoral students. Within the constraints of a limited program duration (within 6 years), most Chinese graduate students operate under extremely high academic pressure. This environment may account for their distinctive understanding and coping approaches to stress. These doctoral students may not prioritize the elimination of Academic Stress; they may even maintain such pressure to sustain academic motivation.

Expressive Suppression is a characteristic trait of East Asian individuals. Compared with their Western counterparts, East Asian individuals may be, to some extent, immune to the negative effects of Expressive Suppression. This is one reason why East Asian individuals employ Expressive Suppression more frequently. Research by Chen et al. (2020) found that when using Expressive Suppression, East Asian individuals experience fewer negative consequences and are more likely to benefit from Expressive Suppression compared to Western individuals. This trait may explain another mechanism identified in this study, namely, “Academic Stress → Expressive Suppression → Academic Achievement.”

The relatively low *R*^2^ values in Models 1, 4, and 6 in Section 3.4 suggest the possible existence of other transformative efforts similar to Emotion Regulation, such as academic engagement or self-efficacy.

This study identified Cognitive Decline as a negative mechanism linking Academic Stress and Subjective Academic Achievement. Beyond Cognitive Decline, academic exhaustion (Lai et al., 2026), learning motivation and depression (Zhang and Kool, 2026) also represent potential negative mechanisms. The relatively low *R*^2^ values in Models 2, 5, and 7 in Section 3.4 indicate that, for the Chinese doctoral students in this sample, there exist other negative mechanisms between Academic Stress and Subjective Academic Achievement not addressed in this study.

Another finding of this study is that Mind Wandering plays a moderating role in the transformation pathways of Academic Stress: it may strengthen the activation of Emotion Regulation by stress, while also exacerbating the erosion of cognitive function by stress. This result resonates with recent discussions on the functional heterogeneity of Mind Wandering, namely, that the effects of Mind Wandering depend on its content, context, and the individual's level of metacognitive monitoring. Why does Mind Wandering produce two opposing moderating effects? This study suggests that this depends on the content valence of Mind Wandering and the level of metacognitive monitoring. When Mind Wandering primarily involves positive and constructive content (e.g., future academic planning, potential solutions to problems), it may stimulate Emotion Regulation motivation; when Mind Wandering primarily involves negative and ruminative content, it is more likely to exacerbate Cognitive Decline. This inference is consistent with the findings of Yamaoka and Yukawa (2020): the impact of Mind Wandering on creativity and mental health depends on its nature. Furthermore, meta-awareness plays a critical role in this process: intentional Mind Wandering may serve goal-directed Emotion Regulation, whereas unintentional Mind Wandering is more likely to become aimless attentional drift (Konjedi and Maleeh, 2017).

### 5.2 Recommendations

This study finds that Academic Stress can stimulate Emotion Regulation motivation, thereby positively influencing Subjective Academic Achievement. Based on this finding, reinterpreting stress as an opportunity for growth rather than a pure threat is key to facilitating stress transformation. It is recommended that university mental health departments develop stress reappraisal intervention programs. The core of this intervention lies in guiding doctoral students, through Cognitive Reappraisal training, to alter their interpretations of stressful events—for example, reframing paper rejection as an opportunity for improvement and viewing dissertation defense pressure as a chance to demonstrate competence rather than a threat to self-worth. Previous research has reached similar conclusions. For instance, Jamieson et al. (2018) found that students who received stress reappraisal interventions performed better on subsequent math tests. Yeager et al. (2022) found that stress reappraisal helped students maintain higher academic persistence.

This study finds that Mind Wandering significantly moderates both pathways: high Mind Wandering can both strengthen the activation of Emotion Regulation motivation by stress and exacerbate the correlation between stress and Cognitive Decline. This suggests that interventions targeting Mind Wandering require nuanced design—preserving its potential positive effects (facilitating Emotion Regulation) while curbing its erosion of cognitive function. Based on this finding, it is recommended that universities introduce mindfulness meditation training, particularly modules focusing on “metacognitive monitoring.” The essence of mindfulness is cultivating non-judgmental awareness of present-moment experiences, which precisely addresses two key dimensions of Mind Wandering: by enhancing metacognitive awareness, it helps individuals recognize when Mind Wandering occurs (Rahl et al., 2017); by training attentional stability, it reduces unintentional, task-unrelated Mind Wandering (Mrazek et al., 2013).

### 5.3 Limitations and future directions

This study employed the Subjective Academic Achievement Scale (Stadler et al., 2021) to measure Academic Achievement, a method with considerable subjectivity. Future research may extend this line of inquiry by incorporating objective indicators such as exam scores.

Although the two waves of questionnaire collection were separated by 4 weeks, this remains insufficient to fully establish strict causal relationships among the variables. Future studies could employ time-series designs to further explore these relationships (Table 5).

**Table 5:** Qualitative interview coding.

Selective coding	Axial coding	Open coding	Excerpts from original data
Cognitive reappraisal	Upward reappraisal	Self-motivation	I once considered giving up. However, the employment rate for my major is relatively low, so I must obtain a doctoral degree.
		Task focus	Academic stress can make me more focused, so I wish to maintain this pressure.
		Expectation	Obtaining a doctoral degree can provide me with good career prospects, so I ultimately chose to persevere.
		Challenge and opportunity	The pressure to publish papers is an opportunity for me to enhance my research capabilities; this mindset gives me more motivation.
	Downward reappraisal	Lowering expectations	I am a university lecturer employed full-time, so I have no job-seeking pressure. There is no need for me to exert excessive effort.
		Downward reconstruction of the meaning of learning	During my doctoral studies, I have already acquired substantial knowledge and skills; academic achievement is not my sole goal.
Expressive suppression	Supervisor-student relationship	Vertical obedience	I am dissatisfied with my supervisor, but I must suppress my dissatisfaction toward him.
		Supervisor's assistance	My supervisor can provide tremendous help for my research, so I must carefully manage my interactions with him.
	Peer relationship	Daily interpersonal relationships	Good interpersonal relationships can contribute to a better campus living environment.
		Team collaboration	I strive to maintain relationships with my peers. Academic collaboration among peers can enhance my academic achievement.

The Achievement-oriented (Upward) Cognitive Reappraisal and Stress Reduction-oriented (Downward) Cognitive Reappraisal proposed in this study represent innovative concepts. The Emotion Regulation Questionnaire developed by Gross and John (2003) focuses on assessing the use of reappraisal strategies but does not evaluate reappraisal orientation, thus offering broad applicability. This study employed qualitative interviews to explore these two types of reappraisal. Future research could develop corresponding subscales to conduct further quantitative investigations.

## Data Availability

The raw data supporting the conclusions of this article will be made available by the authors, without undue reservation.

## Footnotes

1 Chinese Social Sciences Citation Index (CSSCI), the latest version of which indexes over 500 journals across all social science disciplines.
